# Factors influencing incidents of complications while using nickel-titanium rotary instruments for root canal treatment

**DOI:** 10.1186/s12903-019-0938-7

**Published:** 2019-11-11

**Authors:** Ahmad A. Madarati

**Affiliations:** 1 Restorative Dental Sciences Department, College of Dentistry, Taibah University, P.O Box 2898, Madina, 43353 Saudi Arabia; 2 Restorative Dentistry Department, Faculty of Dentistry, Aleppo University, Aleppo, Syria

**Keywords:** Endodontics, NiTi, Rotary, Root canal, Complications, Fracture, Questionnaire, Separated

## Abstract

**Background:**

To investigate the complications associated with the use of nickel titanium rotary instruments (NiTi-RIs) for root canal treatments (RCTs), in Saudi Arabia dental practice, and to explore the influencing factors.

**Methods:**

After obtaining an ethical approval, two pilot studies were conducted to formulate the final questionnaire. The sample size was measured taking into consideration 60% expected response rates and confidence level of 99.9%. The questionnaire was emailed to 600 general dentists (GDs) randomly selected from the dental register and all of the endodontists (175). The email’s introduction clarified objectives of the study and guaranteed that all of the collected information would remain confidential. A reminder was sent after 10 weeks. The data were collected and analyzed using the chi-squared test at a 0.05 significance level*.*

**Results:**

With a 51% overall response rate, 71.9% off the respondents used NiTi-RIs. The majority (83.1%) experienced *complications* while using NiTi-RIs; with the *instruments’ fracture* being significantly the most common complication (52.7%) (*p < 0.001*). The majority (87.7%) experienced *NiTi-RIs’ fracture* *at least once*; with more endodontists (94.3%) than GDs (83.3%) (*p < 0.001*). The greater the *number of weekly performed RCTs* and *participants’ experiences*, the more *NiTi-RIs fractures* and the greater the number of *fracture incidents* (*p < 0.001*). While 60% of those who performed *1–3 RCTs* per week experienced *NiTi-RIs fractures*, 100% of those who performed *more than 12 RCTs* per week did so. The highest percentage of those who experienced *more than 10* fractured NiTi-RIs (60%) was within the group who performed *more than 12 RCTs* per week. Although *fracture incidents* decreased with a smaller number of *reuses,* there was no significant correlation between the *number of fractured inst*ruments and NiTi-RIs *discard strategy* (*p ≥ 0.05*).

**Conclusion:**

Fracture incidence was the most common complication while using NiTi-RIs, regardless of the clinicians’ experiences and skills. While the si*ngle use* may reduce NiTi-RIs fractures, to some extent, the greater *number of RCTs* performed per week was the most influential factor.

## Background

Sufficient debridement and shaping of the root canal systems is one of the most important procedures toward a more predictable root canal treatments (RCTs) outcome [1]. Forty-five years ago, Schielder established the main objectives of cleaning and shaping as follows: cleaning as much as possible, maintaining the canal’s original shape and the apical foramen’s original position, keeping the apical constriction as small as possible, and forming a continuously tapering funnel from the orifice to the apex [2]. Consequently, clinicians should understand the designs and characteristics of the different instruments used for cleaning and shaping. Stainless steel instruments (SSIs), due to their greater fracture resistance, were dominant over carbon steel for many decades [3]. However, they lack flexibility and the ability to maintain the root canal’s original shape and position [4]; therefore, they hinder the debridement of the root canal system [5].

Nickel titanium (NiTi) alloys were developed in the early 1960s, and then, were introduced into dentistry [6]. These alloys can fully recover from as much as 8% strain deformation compared to the less than 1% associated with stainless steel ones (SS) [4, 6]. Moreover, most of NiTi instruments are manufactured by grooving (machining), which allows the production of more instrument designs when compared to SSIs, which are manufactured by twisting [6]. Consequently, manufacturers have produced numerous NiTi root canals’ instruments, especially rotary instruments (RIs).

NiTi-RIs have been reported superior in cleaning and shaping when compared to SSIs [5, 7, 8]. A one-year study, describing 40 cases, showed significantly better outcomes in those cases instrumented using NiTi files when compared to those cases treated with SSIs [5]. Accordingly, they were recommended for faster and safer instrumentation. However, the adoption of NiTi instruments in daily endodontic practice, especially RIs, is still questionable. Some reports have shown different trends in the adoption of these instruments in different countries [9–13]. While only 22% of Australian general dentists (GDs) have been using NiTi-RIs [9], 80% of Swiss dentists have been doing so [10]. Madarati et al. found that 93% of endodontists and 65% of the GDs in the United Kingdom were using NiTi-RIs [11], and another report found that 74% of the GDs in the United States used NiTi-RIs [12]. A systematic review confirmed the different adoption modalities of NiTi-RIs in routine practice and academic sector among different countries [13]. Obviously, with the different practice set ups and different work environments, inconsistent results are expected regarding the use of NiTi-RIs and the associated factors. For example, the literature showed only three studies related to the use of NiTi-RIs in Saudi dental practice [14–16]. Two of them investigated the different modalities and aspects of RCTs procedures, including whether or not NiTi-RIs were used by clinicians [15, 16]. The third study investigated only the uptake of NiTi-RIs and the reasons for using/not using them [14].

Consequently, there is an urgent need to evaluate the important aspects, such as the NiTi-RI reuse strategies, and their relationships to the occurance of complications while cleaning and shaping, especially incidents of instruments fracture. Additionally, reporting complications that clinicians encounter while using NiTi-RIs when compared to hand instruments is paramount. This is especially correct with the fact that the NiTi-RI designs and properties have significantly improved over the last decade [17]. Thus, the following question requires an answer: “what is the percentage of dentists and endodontists who had experienced complications as a results of using NiTi-RIs during root canal cleaning and shaping?”. Such important information could be attained by questionnaire studies [18]; however, they should be well planned and properly conducted in order to produce representative results [18, 19].

The aim of this questionnaire study was to investigate the complications associated with NiTi-RI usage, if any, in Saudi Arabian dental practice and to explore the influencing factors.

## Methods

This study received the ethical approval from the Research Ethics Committee at Taibah University, Saudi Arabia (No 19012014). This research did not require participants’ consent as it was an online study, in which participants’ identities were not required, and their responses were anonymous. The study was conducted in accordance to the World Medical Association’s Helsinki Declaration. Two pilot studies were performed on staff members (Faculty of Dentistry at Taibah University) and two groups of GDs and endodontists (30 and 10, respectively) to formulate the most relevant and easily answered questions, so it reduces the multiple interpretations bias. The final questionnaire (Additional file 1) covered the following main aspects: the participants’ demography and general information on RCTs procedures, NiTi-RIs reuse strategies, complications associated with using NiTi-RIs, and incidence and number of NiTi-RIs fractures and the associated factors. The sample size was calculated taking into consideration the overall number of GDs in Saudi Arabia, a 60% expected response rate and a 48% minimum accepted response rate, and the confidence level. While a 99.9% confidence level resulted in a sample size of 239 GDs, the questionnaire was sent to 600 GDs. This allowed more reliable statistical comparisons among study’s subgroups, because it minimized the number of expected cells which counted less than five. The inclusion criteria were clinicians who are register in the Saudi Dental Register, practicing dentistry in Saudi Arabia and performing RCTs. The 600 GDs were randomly selected using a systematic sampling method [9, 11], and the questionnaire was electronically emailed to the 600 GDs and the 175 endodontists working in Saudi Arabia using the survey tool at http://www.google.co.uk. The email clarified the study’s objectives and confirmed that all of the information provided would remain confidential and that the participants’ identities would remain anonymous. A reminder was sent to all candidates after 10 weeks to encourage those who did not respond to complete the questionnaire. The responses were collected and data were analyzed using IBM SPSS Statistics for Windows version 20.0 (IBM Corp., Armonk, NY, USA) with a chi-squared test at a significance level of 5%.

## Results

### Response rate and participant classification

In total, 395 out of 775 recipients responded to the questionnaire, resulting in an overall response rate of 51% (Non-endodontists response: 298/600: 49.7% and Endodontists response rate: 97/175 = 55.4%). The respondents included 264 (66.8%) GDs, 97 (24.6%) endodontists, 12 (3%) students or residents in endodontic postgraduate programs, and 22 (5.6%) others (other specialties members).

### Non-response Bias and NiTi-RI usage

Overall, 71.9% of the respondents were using NiTi-RIs (*p < 0.001*) (Table 1). While early respondents are defined as those who participated in the study upon the first sent-out, the late respondents are defined as those who participated in the study after the reminder sent-out [9]. There was no significant difference between the percentage of *early respondents* who were using NiTi-RIs (69.9%) and that of the *late respondents* (75%) (*p = 0.172*). Also, there was no significant difference between the percentage of *early respondents* who *experienced complications* while using NiTi-RIs (84.9%) and that of the *late respondents* (80%) (*p = 0.361*). In addition, there was no significant difference between the percentage of *early respondents* who experienced *NiTi-RI fractures* (85.6%) and that of the *late respondents* (90.7%) (*p = 0.214*).

**Table 1:** Details of non-response bias and uptake of NiTi-RIs (%)

Response Stage	Experienced complications		Total	Experience RI fracture		Total
	Yes	No		Yes	No	
Early Response [70]	129 (84.9)	23 (15.1)	152 (100)	131 (85.6)	22 (14.4)	153 (100)
Late Response [75]	87 (80.6)	21 (19.4)	108 (100)	98 (90.7)	10 (9.3)	108 (100)
**Total** [71.9]	216 (83.1)	44 (16.9)	260 (100)	229 (87.7)	32 (12.3)	261 (100)

The values in brackets are the percentage of respondents who were using NiTi-RIs.

### NiTi-RIs’ discard strategies

Significantly, the highest percentage of respondents (37.5%) discarded NiTi-RIs after a *certain number of uses* according to *teeth type* (*p < 0.001*) (Table 2). In *anterior teeth*, while most endodontists discarded NiTi-RIs *after 2–5 RCT* cases (76.7%), most GDs (52.9%) did so after performing *6–10 cases* (*p < 0.001*). In *posterior teeth*, most endodontists (69.8%) adopted the *single-use* policy, but most GDs (64.7%) discarded NiTi-RIs after *2–5 cases*. Moreover, 18.8% of respondents discarded the NiTi-RIs after a *single use* in severely curved canals [significantly more endodontists (27.6%) than GDs (12.5%)].

**Table 2:** Patterns of NiTi-RIs’ Discard Strategy & Associated Factors (%)

Main Discard Strategy		Respondents’ Classification (%)			
		General Dentists	Endodontists	Others	Total
Number of uses according to teeth type		51 (35.4)	43 (41)	4 (33.3)	98 (37.5)
Number of use regardless teeth type		36 (25)	22 (21)	2 (16.7)	60 (23)
When distorted		15 (10.4)	1 (1)	2 (16.7)	18 (6.9)
Period of time regardless teeth type		3 (2.1)	6 (5.7)	0 (0)	9 (3.4)
Single use in severely curved canal		18 (12.5)	29 (27.6)	2 (16.7)	49 (18.8)
Number of uses according to files’ size		9 (6.3)	1 (1)	0 (0)	10 (3.8)
Period of time according to files’ size		3 (2.1)	0 (0)	0 (0)	3 (1.1)
Loss of cutting efficiency		9 (6.3)	3 (2.9)	2 (16.7)	14 (5.4)
Total		144 (100)	105 (100)	12 (100)	261 (100)
Respondents’ Classification	Single case	2–5 cases	6–10 cases	More than 10 cases	Total
Discard Strategy after performing RCTs in Anterior teeth (%)					
General Dentists	0	18 (35.3)	27 (52.9)	6 (11.8)	51 (100)
Endodontists	0	33 (76.7)	10 (23.3)	0 (0)	43 (100)
Others	0	2 (50)	2 (50)	0 (0)	4 (100)
Total	0	53 (54.1)	39 (39.8)	6 (6.1)	98 (100)
Discard Strategy after performing RCTs in Posterior teeth (%)					
General Dentists	9 (17.6)	33 (64.7)	6 (11.8)	3 (5.9)	51 (100)
Endodontists	30 (69.8)	13 (30.2)	0 (0)	0 (0)	43 (100)
Others	2 (50)	2 (50)	0 (0)	0 (0)	4 (100)
Total	41 (41.8)	48 (49)	6 (6.1)	3 (3.1)	98 (100)

### NiTi-RIs complications versus hand files

The majority (83.1%) experienced *complications* while using NiTi-RIs, with no significant difference between endodontists and GDs (*p* = 0.370) (Table 3). Significantly, a *fracture of NiTi-RIs* was the most common complication (52.7%) [*p < 0.001*]. Most respondents (67.7%) reported that *NiTi-RIs* caused *less complications* when compared to *hand instrument*s (*p < 0.001*), with a significantly greater percentage of endodontists (78.8%) than that of GDs (60.4%) [*p < 0.001*].

**Table 3:** Complications of NiTi-RIs usage and comparisons with other instruments’ types (%)

Complications of NiTi-RIs Use	Respondents’ Classification (%)				
	General Dentists	Endodontists	Others	Total	Cumulative Total
Ledge formation	18 (12.5)	7 (6.7)	1 (8.3)	26 (10)	26 (10)
Root perforation	3 (2.1)	2 (1.9)	1 (8.3)	6 (2.3)	6 (12.3)
Straightening of canals	9 (6.2)	9 (8.7)	0 (0)	18 (6.9)	18 (19.2)
Files wedging	3 (2.1)	3 (2.9)	0 (0)	6 (2.3)	6 (21.5)
Canals’ transportation	3 (2.1)	6 (5.8)	1 (8.3)	10 (3.8)	10 (25.4)
Post-operation pain	9 (6.2)	3 (2.9	1 (8.3)	13 (5)	13 (30)
Instruments fracture	72 (50)	59 (56.7)	6 (50)	137 (52.7)	137 (83.1)
No complications	27 (18.8)	15 (14.4)	2 (16.7)	16 (16.9)	260 (100)
Total	144 (100)	104 (100)	12 (100)	260 (100)	
Respondents’ Classification	Which Instruments Caused More Complications (%)				
	NiTi-RIs	SS hand files	The Same rate	Never had complications	Total
General Dentists	18 (12.5)	87 (60.4)	30 (20.8)	9 (6.2)	144 (100)
Endodontists	1 (1)	82 (78.8)	12 (11.5)	9 (8.7)	104 (100)
Others	1 (8.3)	7 (58.3)	2 (16.7)	2 (16.7)	12 (100)
Total	20 (7.7)	176 (67.7)	44 (16.9)	20 (7.7)	260 (100)

### NiTi-RI fracture incidents and associated factors

The majority (87.7%) experienced *NiTi-RIs fractures* at least once since they started using them (*p < 0.001*), with more endodontists than GDs (94.3 and 83.3%, respectively) [*p = 0.009*] (Additional file 2: Table S1). The percentage of respondents who experienced *NiTi-RIs fractures* while working in the *academic* sector (71.4%) was lower than those who were working in the *private* and *governmental* sectors (91.4 and 86%, respectively) [*p = 0.027*] (Additional file 2: Table S1). Moreover, there was a significant correlation between respondents’ *experience* and the *NiTi-RIs’* *fracture* incidence (*p < 0.001*). The *less experienced* the respondents, the lower the incidents of NiTi-RIs’ fracture; with significantly the lowest percentage among those with *less than 3 years*’ experience (54.5%) [*p < 0.001*]. As the *number of RCTs* performed per week increased, the NiTi-RIs *fracture* incidence increased (*p < 0.001*). Moreover, 60% of those who performed *1–3 RCTs* per week experienced NiTi-RIs fractures, while 100% of those who performed *more than 12 RCTs* per week did so (*p < 0.001*) (Additional file 2: Table S1).

Significantly most respondents (77.6%) experienced less NiTi-RIs fractures *more recently* when compared to when they used them during the *early stages* (*p < 0.001*).

### Comparisons of fracture of different instruments

Significantly, most of the respondents (65.5%) experienced *NiTi-RIs* fractures more often than in other types of instruments; with more endodontists (76.8%) than GDs (57.5%) *[p = 0.011]* (Table 4). In contrast, the percentage of GDs who experienced *SS hand* files fractures more often than *other types* (27.5%) was significantly greater than that of endodontists (14.1%).

**Table 4:** Comparisons of fracture incidents among different instruments type (%)

Respondents’ Classification	Which instruments fractured more? (%)				Total
	NiTi rotary	SS hand files	NiTi hand files	Same fracture rate	
General Dentists	69 (57.5)	33 (27.5)	0 (0)	18 (15)	120 (100)
Endodontists	76 (76.8)	14 (14.1)	3 (3)	6 (6.1)	99 (100)
Others	5 (50)	4 (40)	0 (0)	1 (10)	10 (100)
Total	150 (65.5)	51 (22.3)	3 (1.3)	25 (10.9)	229 (100)

### Number of Fractured NiTi-RIs & Influencing Factors

#### Respondents’ experiences and classifications

There was a significant correlation between both of *respondents’ experience* and *classification* and the *number of NiTi-RIs fractured* in their practice (*p < 0.001*). While most endodontists experienced fractures of *more than 10* NiTi-RIs (52.5%), the highest percentage of GDs experienced fractures of *3–5* instruments (30%) [*p < 0.001*] (Table 5). Most of the participants who had *up to 3 years of experience* (61.1%) encountered *3–5* fractured NiTi-RIs, while the highest percentage of those who had *more than 15 years of experience* (40.9%) encountered *more than 10* fractured NiTi-RIs (*p < 0.001*).

**Table 5:** Frequency of factors associated with the number of fractured NiTi-RIs (%)”

Respondents’ Classification	Number of fractured NiTi-RIs (%)				Total
	1–2 files	3–5 files	6–10 files	More than 10 files	
General Dentists	24 (20)	36 (30)	33 (27.5)	27 (22.5)	120 (100)
Endodontists	16 (16.2)	17 (17.2)	14 (14.1)	52 (52.5)	99 (100)
Others	2 (20)	4 (40)	4 (40)	0 (0)	10 (100)
Total	42 (18.3)	57 (24.9)	51 (22.3)	79 (34.5)	229 (100)
Respondents’ Experience	Number of fractured NiTi-RIs (%)				Total
	1–2 files	3–5 files	6–10 files	More than 10 files	
Up to 3 Years	0 (0)	11 (61.1)	4 (22.2)	3 (16.7)	18 (100)
3.1 to 7 Years	16 (25.4)	22 (34.9)	14 (22.2)	11 (17.5)	63 (100)
7.1 to 15 Years	15 (18.3)	6 (7.3)	23 (28)	38 (46.3)	82 (100)
More Than 15 Years	11 (16.7)	18 (27.3)	10 (15.2)	27 (40.9)	66 (100)
Total	42 (18.3)	57 (24.9)	51 (22.3)	79 (34.5)	229 (100)
Weekly Performed RCTs	Number of fractured NiTi-RIs (%)				Total
	1–2 files	3–5 files	6–10 files	More than 10 files	
1–3 cases	9 (33.3)	8 (29.6)	4 (14.8)	6 (22.2)	27 (100)
4–6 cases	15 (24.6)	23 (37.7)	16 (26.2)	7 (11.5)	61 (100)
7–12 cases	8 (8.3)	22 (22.9)	27 (28.1)	39 (40.6)	96 (100)
More than 12 cases	10 (22.2)	4 (8.9)	4 (8.9)	27 (60)	45 (100)
Total	42 (18.3)	57 (24.9)	51 (22.3)	79 (34.5)	229 (100)
Number of fractured instruments	Duration of Using NiTi-RIs (%)				Total
	1–2 files	3–5 file	6–10 files	More than 10 files	Total
Up to I month	3 (100)	0 (0)	0 (0)	0 (0)	3 (100)
6 months	1 (100)	0 (0)	0 (0)	0 (0)	1 (100)
One year	9 (33.3)	9 (33.3)	3 (11.1)	6 (22.2)	27 (100)
3 years	18 (37.5)	15 (31.3)	9 (18.8)	6 (12.5)	48 (100)
More than 3 years	11 (7.3)	33 (22)	39 (26)	67 (44.7)	150 (100)
Total	42 (18.3)	57 (24.9)	51 (22.3)	79 (34.5)	229 (100)

#### Number of RCTs performed weekly

As the *number of weekly performed RCTs* increased, the number of *fractured NiTi-RIs* increased (*p < 0.001*). The highest percentage of those who experienced *more than 10* fractured NiTi-RIs (60%) was within the group who performed *more than 12 RC*Ts per week (Table 5).

#### Experience in using NiTi-RIs

The l*onger* the participants had been using NiTi-RIs, the *greater the number* of fractured instruments they experienced (*p < 0.001*) (Table 5). The percentage of those who experienced *more than 10* fractured files increased from 0% among those who used the NiTi-RIs for *up to 6 months* to 7.6 and 84.8% among those who used them for *three* and *more than three year*s, respectively (*p < 0.001*).

#### NiTi-RIs discard strategy

There was no correlation between the *number of fractured* NiTi-RIs and *instruments discard strategy* (*p ≥ 0.05*). However, the percentage of those who fractured *more than 10* files and discarded files after *a single use* (40%) was lower than that of those who experienced the same number of fractured files, but did not discard files after *a single use* (44.2%) [*p = 0.578*] (Table 5).

## Discussion

In Saudi Arabia, routine usage of NiTi-RIs is implemented in the endodontic postgraduate programmes’ curricula (Personal communication). In addition, the majority of cases performed during the residency and postgraduate endodontic programmes, if not all, are RCTs cases. Hence, endodontic postgraduate students or residents were classified as endodontists. In addition, this enabled better statistical comparisons among subgroups. This study investigated the possible correlation of modalities of NiTi-RIs usage and the complications may be encountered during their usage. The study results revealed that fracture of NiTi-RIs was the most common complication. One possible contributing factor is the number of clinical uses of NiTi-RIs. There has been no agreement on the exact number of NiTi-RIs reuses [20–23]. Similarly, questionnaire studies have also shown different preferences regarding NiTi-RIs uses, although the majority of these studies’ respondents discarded them after a certain number of uses: 56% [24], 84% [9], 70% [11], and 50% [25]. The current study was not an exception; the majority of clinicians discarded NiTi-RIs after a certain number of uses. However, these studies also reported different clinician preferences regarding the number of uses: approximately 62% for up to 6–10 uses [24], 70% for up to 2–5 uses [9], 26% after 6 or more than 6 uses [11], and after 1–4 patients for the majority [25]. These variationns may have been due to the different clinical skills, instruments’ properties, designs, and root-canals’ cleaning and shaping protocols. However, the morphological variations in the root canal systems, especially among different races, are important factors [26]. For example, 70% of Australian dentists reported that their decision about the number of uses was based on anatomical factors [9]. Within the same regard, there is no agreement among NiTi-RIs manufacturers recommendations on specific number of uses, due to the different designs, specifications and manufacturing processes. Moreover, manufacturers usually are not definite when recommending number of uses, except for those NiTi-RIs that are specially designed and manufactured for a single use. In addition, it should be noted that fracture of NiTi-RIs is a multifactorial incident, so many factors may contribute to failure of NiTi-RIs including: number of clinical uses, root canals anatomy, rotation speeds and torques used while instrumentation, sterilization of NiTi-RIs and others [20–23]. The current study was the first one to reflect on these influencing factors, in that it specified a certain number of reuses based on the tooth types, cases difficulty, and files sizes. The results showed clearly lower usage frequencies when NiTi-RIs were used in posterior teeth. In anterior teeth, while most endodontists (76.7%) discarded NiTi-RIs after 2–5 cases, most GDs (52.9%) did so after 6–10 cases. Contrarily, in posterior teeth, whilst most endodontists (69.8%) adopted the single use policy, most of GDs (64.7%) discarded NiTi-RIs after 2–5 cases. Moreover, 18% discarded NiTi-RIs after using them in severely curved canals. Nevertheless, this wide range of preferences for the reuse numbers as well as the multiple influencing factors mentioned above, gave rise to a single-use policy. Studies have shown better instruments’ performance when they are discarded after a single use [22, 27, 28]. Despite this fact, the results of previous studies indicate that clinicians, especially GDs, still need to adopt this policy. While none of the respondents to the study by Mozayeni et al. discarded files after a single use [24], 12 and 44% did so in two other studies [9, 11]. Moreover, none of clinicians in the current study discarded NiTi-RIs after a single use in anterior teeth; however, 41.8% did so in posterior teeth. Only Madarati et al. reported a relatively higher percentage of respondents (44%) in the United Kingdom that adopted a single-use policy [11]. This could be due to the strong recommendation for the single use of NiTi-RIs in order to minimize the risk of Creutzfeldt-Jakob disease transmission [29]. However, it is worth mentioning that a single-use policy may create some economic pressure on clinicians [29]. Nevertheless, like most previous studies, endodontists in the current study showed better awareness than GDs regarding the importance of using NiTi-RIs for less cases. Endodontists are usually exposed to more literature during their postgraduate training programmes. In addition, they usually have to deal with more difficult and complex cases that necessitate a single-use policy.

The results of the current study were consistent with those of previous studies. In that, the majority of respondents (83.1%) experienced one or more procedural errors while using NiTi-RIs [9, 24]. These findings confirm the fact that procedural errors during cleaning and shaping are common. However, it is well-accepted that using NiTi-RIs has contributed to lower complication frequencies, to some extent, when compared to SSIs. A meta-analysis study found that NiTi-RIs were associated with lower root-canals’ transportation and apical extrusion when compared to SSIs [8]. Similarly, most of the respondents to the current study (67.7%) reported that NiTi-RIs caused less complications than SSIs. This trend was also reported by Mozayeni et al. [24]. Moreover, 90% of the Australian clinician respondents reported that NiTi-RIs preparation is more beneficial than SSIs [9]. Mainly, these findings can be explained by the super-elasticity of NiTi alloy when compared to the SS alloy, which allows 8% greater deformation recovery for NiTi alloy than that of the SS one [4].

Unlike previous studies, this questionnaire asked the participants to specify which complication they encountered most often. The lowest percentages (3.8 and 6.9%) reported that NiTi-RIs associated with canals’ transportation and straightening of root canals (respectively). The second most common complication of NiTi-RIs usage was ledge formation, though low percentage reported it (10%). These low incidents could be attributed to the good flexibility of these instruments. On the other hand, most of the respondents reported fracture of NiTi-RIs as the most common complication (53%). It should be noted that the response to the question was optional. However, the participants had to answer a separate question (answer is mandatory) regarding the incidence of NiTi-RIs fracture. The majority (87.7%) experienced NiTi-RIs’ fractures at least once since they started using them. These figures triggered the author to analyze this aspect and the possible influencing factors. These figures were similar to those obtained by Madarati et al. (88.8%) [11], but greater than those reported by Parashos & Messer and Barbakow & Lutz (74 and 76%, respectively) [9, 10]. The differences might be explained by the different NiTi-RIs used and different clinical skills. Nevertheless, these findings clearly show that introducing the NiTi alloy did not overcome the problem of instruments fracture as some manufacturers claim. When the participants were asked which type of instruments fractured more, most of them (65.5%) experienced NiTi-RIs’ fractures more than any other type of instruments. These results are consistent with those obtained by Madarati et al [11]. Interestingly, more endodontists (94.3%) experienced NiTi-RIs fractures when compared to GDs (83.3%). These findings were similar to those obtained by Madarati et al, and they can be explained by the same reasoning that they indicated [11]. First, more endodontists used NiTi-RIs when compared to GDs (96.9 and 60%, respectively); the more often that instruments are used, the greater the possibility of instruments fracture [11]. Second, endodontists usually deal with more complex cases that GDs opt out of for referral to endodontists. Third, like previous studies [11], this study showed that the greater the number of weekly performed RCTs, the greater the number of instruments’ fracture incidents. Endodontists perform greater numbers of RCTs when compared to GDs. Unsurprisingly, all of those participants who performed more than 12 RCTs, experienced NiTi-RIs fracture incidents at least once. In this respect, the correlation between the NiTi-RIs fractures and the participant’s experience can be explained. Unexpectedly, the current study showed that the less experienced the respondent, the lower the incidence of NiTi-RIs fractures; with the significantly lowest percentage among those who had less than 3 years of experience (54.5%). However, two previous studies did not find correlations between participants’ experience and the fracture incidence [9, 11]. One possible reason was that participants were asked about the fractures of all instruments (hand and rotary), while the current study was only concerned with NiTi-RIs fractures. Another reason might be the different instruments that the participants were using. Again, and as mentioned above, these figures can be explained in light of the number of weekly performed cases. The lowest number of weekly performed cases was among those clinicians with less experience. This could also explain the lower NiTi-RIs fracture incidence among academic respondents and the greater incidences among those working in the private and governmental sectors. However, training and experience in using NiTi-RIs can still influence the fracture incidence. Most of the respondents, in the current study, reported greater NiTi-RIs fracture incidences during the early stage of using them. Like any new technology, mastering the use of NiTi-RIs with minimal errors requires training and additional practice.

Further analysis of the data related to the number of fractured NiTi-RIs since participants began using them would reveal the real impact of factors influencing the fracture incidence. The results again confirmed the great impact of the number of RCT cases performed per week over the other factors, such as participant’s experience since graduation, their classifications (GDs or endodontists), and experiences in using NiTi-RIs. Most of those who experienced more than 10 fractured NiTi-RIs (60%) were within the group who performed more than 12 RCTs per week. As mentioned earlier, and as this study showed, endodontists usually perform more RCTs, especially retreatment and anatomically difficult cases, than GDs do. Therefore, unsurprisingly, most endodontists experienced more than 10 fractured NiTi-RIs. However, the highest percentage of GDs (30%) experienced only 3–5 fractured NiTi-RIs. For the same reason, the greater number of RCTs performed per week also explained why a greater number of fractured instruments was reported by those who had more years of experience. While most of those participants who had up to 3 years of experience (61.1%) encountered 3–5 fractured files, the highest percentage of those who had more than 15 years’ experience (40.9%) encountered more than 10 fractured NiTi-RIs. This could also explain why the number of fractured NiTi-RIs increased among those who had been using them for more than three years. Nevertheless, the impact of precautionary procedures that may reduce instruments’ fractures should not be neglected, and clinicians still need to do their best to minimize their occurrence. The current study confirmed this fact, and showed a lower number of fractured NiTi-RIs among those who discarded files after a single use when compared to those who did not adopt this policy. Previous studies reported lower fracture incidences when NiTi-RIs were only used once [22, 27, 28]. Nevertheless, and as indicated earlier, instruments fracture, especially NiTi-RIs fracture is a multifactorial incident, so many factors may contribute to failure of NiTi-RIs including: number of clinical uses, root canals anatomy, manufacturing proccess, sterilization of NiTi-RIs, instrumentation conditions, and others [20–23, 30, 31]. This may explain, to great extent, some discrepancies within the results of the current study as well as the previous ones.

This study achieved an overall response rate of 51%, which may be low and can be considered as one main limitation, especially when compared to those obtained in previous studies [9, 32]; 70–80% is usually preferred in questionnaire studies [33]. However, certain factors and facts should be considered that justify and rebut this low response rate. The lowest level of non-response bias could have been obtained with a 43% response rate [34]. Also, it is well-accepted that the response rates obtained in web-based questionnaire studies are usually lower than those reported in self-administrated ones [35]. In addition, low response rates with random and systematic sampling are better than high response rates without randomization [19]. Moreover, one main factor that can minimize the response bias is easily-answered questions that prevent multiple interpretations [19]. The current study was conducted following two pilot studies that assured the most relevant and well-understood questions. Last but not least, the crucial measure to validate questionnaire studies results is to make sure that there is no significant difference between those who responded to the questionnaire and those who did not. This can be achieved statistically by comparing those who responded after the first questionnaire sent out (early responses) and those who responded after the reminder (late responses), because the latter represent those who did not respond to the questionnaire [9, 11]. There was no significant difference between the percentage of *early and late respondents* who were using NiTi-RIs (69.9 and 75%, respectively) (*p = 0.172*). Also, there was no significant difference between the percentage of *early* respondents who *experienced complications* while using NiTi-RIs (84.9%) and that of the *late* respondents who did so (80%). Additionally, there was no significant difference between the rate of the *early* respondents who experienced *NiTi-RI fractures* (85.6%) and that of the *late* respondents (90.7%).

## Conclusions

Within the limitations of this study, it can be concluded that NiTi-RIs’ fracture was the most common mishape associated with NiTi-RI usage, regardless of the clinician’s experience and skills. While a single-use discard policy can reduce the possibility of NiTi-RIs fracture, to some extent, the greater number of weekly performed RCTs was the most influential factor.

## Supplementary information

**Additional file 1:** Endodontic Rotary Systems Use Survey Form.

**Additional file 2: Table S1.** Incidents of NiTi-Fracture and associated factors (%).

## Data Availability

The datasets used and/or analysed during the current study are available from the corresponding author on reasonable request.

## Abbreviations

GDs: General dentists
NiTi-RIs: Nickel titanium rotary instruments
RCTs: Root canal treatments
SSIs: Stain less steel instruments
