# Supplementary material for: Factors influencing incidents of complications while using nickel-titanium rotary instruments for root canal treatment
Source: BMC Oral Health. 2019 Nov 11;19:241. doi: 10.1186/s12903-019-0938-7 (PMC6849290; doi:10.1186/s12903-019-0938-7)
Supplement: Supplementary file 1 — Additional file 1: Endodontic Rotary Systems Use Survey Form. [file 12903_2019_938_MOESM1_ESM.docx]

**Endodontic Rotary Systems Use Survey Form**

**How long have you been practicing dentistry?**

Up to 3 years

3.1 to 7 years

7.1 to 15 years

More than 15 years

**Where do you work basically?**

Private Centre or Hospital

Academic

Governmental Hospital or Centre

Postgraduate studies programme

**Are you?**

General Dental Practitioner (GDP)

Endodontist

Resident or postgraduate student in endodontics Other:

**On average, how many root canal treatments (RCT) do you perform PER WEEK?**

Never do RCT *.*

1-3 cases

4-6 cases

7-12 cases More than 12

**Are you CURRENTLY using nickel titanium (NiTi) rotary system(s) for root canal preparation? ***

Yes

No

**Over what period of time you have been using NiTi rotary files?** *Mark only one oval.*

Up to one month

6 months

One year

3 years

More than 3 years

**What is your MAIN strategy for discarding (stop using) NiTi rotary files?** *Mark*

After certain number of use ACCORDING to Teeth Types

After certain number of use REGARDLESS teeth types

When the file is deformed (distorted) *.*

After certain period of time ACCORDING to teeth types

After certain period of time REGARDLESS teeth types *.*

After a single use in severely curved or very narrow canals

After certain number of use ACCORDING to the size of instruments

After certain period of time ACCORDING to the size of instruments

When the file(s) loses its cutting efficiency

**For how many times (cases) do you use a NiTi rotary file?**

Single use

2 to 5 cases

6 to 10 cases

More than 10 cases

**For how many times (cases) do you use a NiTi rotary file in Anterior Teeth including premolars with straight or slightly curved canals?***.*

Single use

- 1. to 5 cases

6 to 10 cases

More than 10 cases

**For how many times (cases) do you use a NiTi rotary file in Posterior Teeth, including premolars with severely curved canals?**

Single use

- 1. to 5 cases

6 to 10 cases

More than 10 cases

**For how long do you use a NiTi rotary file?**

1-3 days

4-6 days

2 weeks

3-4 weeks

More than 4 weeks

**For how long do you use a NiTi rotary file in Anterior Teeth, including premolars with straight or slightly curved canals?**

1-3 days

4-6 days

2 weeks

3-4 weeks

More than 4 weeks

**For how long do you use a NiTi rotary file in Posterior Teeth, including premolars with severely curved canals?***.*

1-3 days

4-6 days

2 weeks

3-4 weeks

More than 4 weeks

**For how long do you use Small-size rotary files?**

1-3 days

4-6 days

2 weeks

3-4 weeks

More than 4 weeks

**For how long do you use Medium-size rotary files?**

1-3 days

4-6 days

2 weeks

3-4 weeks

More than 4 weeks

**For how long do you use Large-size rotary files?**

1-3 days

4-6 days

2 weeks

3-4 weeks

More than 4 weeks

1. **For how many times (cases) do you use Small-size rotary files?**

Single use

- 1. to 5 cases

6 to 10 cases

More than 10 cases

1. **For how many times (cases) do you use Medium-size rotary files?**

Single use

- 1. to 5 cases

6 to 10 cases

More than 10 cases

1. **For how many times (cases) do you use Large-size rotary files?**

Single use

- 1. to 5 cases

6 to 10 cases

More than 10 cases

**Have you ever experienced fracture of NiTi rotary files SINCE you started using them? ***

Yes

No

**How many NiTi rotary files, roughly, have been fractured since you started using them?**

1-2 files

3-5 files

6-10 files

More than 10 files

**Compared to the early stage (when started using rotary systems), instruments fracture rate**

More fracture recently compared to the early stage

Less fracture recently compared to the early stage

Instruments fracture rate is nearly the same for both the early and recent stage

**Compared to canal preparation using only Hand instruments, which instruments fractured more?**

NiTi rotary files

SS Hand files

NiTi Hand files

Instruments fracture rate is almost the SAME for all

# Complications during canal preparation

**What is the MOST common complication (mishap) you have encountered since you started using NiTi Rotary files?** *Mark only one oval.*

Ledge formation

Root Perforation, including Stripping perforation *.*

Weakened roots (excessive dentine removal)

Straightening of curved canals

Wedging (interlocking) of a file in the canal *.*

Transportation of the canal

Post-cleaning and shaping pain

Intra-canal fracture of Rotary Files

I never had any complications with rotary files

# Complications comparison

**Compared to canal preparation with HAND instruments only;**

(Instruments fracture is not included) *Mark only one oval.*

You have experienced MORE complications with rotary files

You have experienced LESS complications with rotary files

Complications rate is almost the SAME with both types of files

You have never experienced complications during canal preparation
