# Supplementary material for: Factors influencing incidents of complications while using nickel-titanium rotary instruments for root canal treatment
Source: BMC Oral Health. 2019 Nov 11;19:241. doi: 10.1186/s12903-019-0938-7 (PMC6849290; doi:10.1186/s12903-019-0938-7)
Supplement: Supplementary file 2 — Additional file 2: Table S1. Incidents of NiTi-Fracture and associated factors (%). [file 12903_2019_938_MOESM2_ESM.docx]

|  | **Professional** **Classification (%)** | | | | | | | **Place of Work (%)** | | | | | | |  |  |
| --- | --- | --- | --- | --- | --- | --- | --- | --- | --- | --- | --- | --- | --- | --- | --- | --- |
|  | **GDs** | **Endodontists** | | | | **Others** | | **Private** | | **Government** | | | **Academic** | |  |  |
| **Experience of Instruments Fracture** | 120 (83.3) | 99 (94.3) | | | | 10 (83.3) | | 128 (91.4) | | 86 (86) | | | 15 (71.4) | |  |  |
| Total | 229 (87.7) | | | | | | | 229 (87.7) | | | | | | |  |  |
|  | **Experience of Respondents (Years) (%)** | | | | | | | | | | | | | |  |  |
|  | **Up to 3** | | | **3.1 to 7** | | | | **7.1 to 15** | | | | **More than 15** | | |  |  |
| **Experience of NiTi-RIs Fracture** | 18 (54.5) | | | 63 (94) | | | | 82 (92.1) | | | | 66 (91.7) | | |  |  |
|  | 81 (81) | | | | | | | 148 (91.9) | | | | | | |  |  |
| **Total** | **229 (87.7)** | | | | | | | | | | | | | |  |  |
|  | **Number of cases performed per week (%)** | | | | | | | | | | | | | |  |  |
| **Experience of NiTi-RIs fracture** | **1-3 cases** | | **4-6 cases** | | | | **7-12 cases** | | | | **More than 12 cases** | | | |  |  |
|  | 27 (60) | | 61 (89.7) | | | | 96 (93.2) | | | | 45 (100) | | | |  |  |
| **Total** | 229 (87.7) | | | | | | | | | | | | | |  |  |
|  | **Fracture rate at different stages of use (%)** | | | | | | | | | | | | | **Total** |  |  |
| **Respondents’ Classification** | **More fracture recently compared to early stage** | | | | **Less fracture recently compared to early stage** | | | | **Fracture rate is the same** | | | | |  |  |  |
| **General Dentists** | 12 (10.3) | | | | 96 (82.1) | | | | 9 (7.7) | | | | | 117 (100) |  |  |
| **Endodontists** | 6 (6.3) | | | | 72 (75) | | | | 18 (18.8) | | | | | 96 (100) |  |  |
| **Others** | 0 (0) | | | | 5 (50) | | | | 5 (50) | | | | | 10 (100) |  |  |
| **Total** | 18 (8.1) | | | | 173 (77.6) | | | | 32 (14.3) | | | | | 223 (100) |  |  |

**Table 4** Incidents of NiTi-Fracture and associated factors (%)
